# Managing unresolved issues of addiction during cancer treatment: A qualitative study about cancer care providers’ representations

**DOI:** 10.1371/journal.pone.0242693

**Published:** 2020-11-24

**Authors:** Elise Verot, Véronique Regnier Denois, Corinne Macron, Franck Chauvin

**Affiliations:** 1 Centre Hygée, HESPER EA 7425, Université de Lyon, Université de Saint-Etienne, Saint-Etienne, France; 2 Centre Hygée, Institut de Cancérologie Lucien Neuwirth, Saint-Etienne, France; Centre Hospitalier Regional Universitaire de Tours, FRANCE

## Abstract

**Objective:**

Five French oncology institutions had participated in a funded study aiming at implementing an Evidence-Based Practice tool (PAM-13), which allowed nurses to measure the level of activation of the patient to support his or her own empowerment in the cancer care pathway. The purpose of this ancillary study is to (i) describe the caregivers’ perceptions of addictions and their management concurrently with cancer treatment, (ii) explore the role that Motivational Interviewing techniques can play.

**Methods:**

15 individual semi-structured interviews of caregivers were performed, using the Theoretical Domains Framework for the interview guide. Qualitative data were analyzed inductively, in a thematic analysis. The COREQ guided the reporting of this qualitative study.

**Results:**

Views on addictions influence the way caregivers manage patients suffering from unresolved issues of addiction. Care is mainly focused on the pathology (cancer-centered) and strictly curative. When practiced, Motivational Interviewing is patient-centered, fostering the patient’s empowerment on the cancer care pathway.

**Conclusions:**

The dissemination of Motivational Interviewing techniques in current practices in oncology, both in terms of doctors and nursing teams, would enable improvement to the management of addictions on the cancer care pathway, by deploying a patient-centered approach. This new paradigm of care would support the empowerment of patients enrolled in the cancer care pathway and promote better communication between caregivers and patients. Hence, a paradigm shift is essential. Motivational Interviewing techniques could provide a caring approach that promotes communication between the patient and the caregiver and also supports the former’s empowerment. This research suggests the need to adapt the cancer care pathway in order to integrate the necessary care for patients who concomitantly suffer from unresolved addictive disorders.

**Trial registration:**

NCT03706937

## 1 Introduction

France has turned its health policy towards a patient-centered care strategy. The National Health Strategy advocates placing the patient back at the heart of care, with a particular interest in patient experience and feedback, to enable the individual to play an active role in his or her own care [1, 2]. This paradigm shift goes against the paternalistic traditions of French care. The engagement of patients with chronic diseases has been a sought-after goal for many years [3]. Specifically, the French Cancer Plan of 2014–2019 recommended systematic support for smokers who attempt to quit while in cancer treatment facilities [4]. Benarous et al. explained that Motivational Interviewing (MI) has proven it supports and helps encourage motivation of patients to manage themselves in terms of alcohol and tobacco addiction, also by giving positive results on therapeutic adherence [5]. Regarding smoking cessation, Perriot et al. showed that a behavioral approach was more efficient (RR = 2,17; IC95%: 1,52–3,11) than a simple advisory one (RR = 1,24; IC95%: 1,16–1,33) or a total lack of advice [4]. They suggested that, as soon the cancer diagnosis was given, caregivers proposed support intervention for unresolved addictive disorders. Motivational interviewing techniques, combined with medication, turned out to ensure prolonged abstinence [4]. With this two-pronged approach, patient engagement in the cancer care pathway could be made easier [4]. McCarley stated that MI provides effective engagement among patient and care providers, in a collaborative relationship centered on patient goal-setting and self-management [6]. Miller and Rollnick define MI as “a style of collaborative conversation that strengthens a person’s own motivation and commitment to change” [7].

The question of addiction management during cancer care through a motivational approach, using Motivational interviewing techniques, is worth raising. In this paper, we considered the dependence on tobacco, alcohol and illicit drugs in terms of addiction. It should be noted that verbatim interviews that focused on patients with opioid addiction did not include patients with a higher opioid tolerance as a result of cancer pain management.

According to the French National Health Ministry, the consumption of psychoactive substances (alcohol, tobacco, illicit drugs) was responsible for 100,000 deaths per year, caused either by accident or illness. Of these 100,000 deaths, 40,000 were attributable to cancer. Research also shows that addictive behavior was prevalent in 30% of deaths of persons under age 65 [8]. In the spring of 2018, the “onco-addiction group” was created within the French comprehensive cancer centers network, which is a first in France and internationally [9]. Thus, while conducting research at five French comprehensive centers aimed at implementing an Evidence-Based Practice (EBP) tool in nursing practices (in order to foster patient empowerment in the cancer care pathway) [10], we believed it was relevant to undertake an ancillary research study on the subject, given its novelty in France. Indeed, the orientation of the new French health policy recognized prevention and patient experience as two essential elements in the cancer care pathway. However, the recent results of the Compare Cohort demonstrated the persistent gap between expectations, needs expressed by patients and the representations made by healthcare professionals [11].

We therefore sought to question the continuous stigma that plagues those dealing with addiction problems while being treated by healthcare workers, and the latter’s role-related perceptions in such a context. Moreover, we aimed to demonstrate how motivational interviewing techniques could improve patient care.

The aim of this qualitative study is to (i) describe caregivers’ perceptions of addictions and how they manage them concurrently with cancer treatment (ii) explore the role that motivational interviewing techniques can play.

## 2 Methods

A qualitative study was carried out in a French public comprehensive cancer facility situated in a district heavily affected by cancer. The choice to perform a qualitative study was based on the potential that this type of research could potentially contribute to understanding a problem in various dimensions or to study phenomena not yet detected [12]. The Hygée Centre, located in the same area, is a facility of regional resources for cancer prevention, information and education.

### 2.1 Characteristics of the sample

#### 2.1.1 Source and method of recruitment

After meeting with the Administrative Director of nurses and caregivers at the different facilities, the former provided a list of names of all the nurses’ managers to contact in each care unit (oncology and hematology). Each healthcare manager presented the study to his/her entire team, supported by the study information leaflet at their disposal, which had been validated by the Ethics Committee. Depending on the dates of meetings that we had arranged beforehand, the study was proposed to all the professionals who were scheduled to work in the units on these specific days. Concerning the treatment teams, caregivers were contacted either directly by the representatives, who personally arranged the appointments, or through an e-mail sent by the representative, with a copy of the e-mail to our team, requesting they make an appointment with the interviewers.

#### 2.1.2 Inclusion and exclusion criteria

The inclusion criterion consisted of an agreement by the nurses to participate in collecting data. Consent was given by e-mail or verbally during the appointment arrangement process. The exclusion criterion was a refusal to take part in the study. All those designated agreed to participate and were subsequently interviewed.

### 2.2 Semi-structured interviews

#### 2.2.1 Using the Theoretical Domains Framework (TDF)

The TDF was developed by Michie et al. to understand health professionals’ practices and identify elements of these practices to target for the implementation of new recommendations and evidence-based practice [13, 14]. According to Lou Atkins et al., this framework was primarily used in healthcare settings to explore factors influencing clinical behaviors in order to design implementation interventions; to identify barriers and facilitators to change [15–17]. Alan Glasper and Colin Rees explained that each domain of the TDF represented a behavioral determinant, mediators of behavioral change. [18] The TDF was composed of 12 original theoretical domains: (1) knowledge, (2) skills, (3) social/ professional role and identity, (4) beliefs about capabilities, (5) beliefs about consequences, (6) motivation and goals, (7) memory, attention and decision processes, (8) environmental context and resources, (9) social influences, (10) emotion regulation, (11) behavioral regulation, and (12) nature of the behavior. [14] Employing this tool helped identify elements that either hindered or facilitated the implementation of new modes of care in everyday practice. Michie et al. suggested a prompt containing the wording of a series of follow-up questions related to each theoretical area [14]. We performed a double translation of the prompt. We then conducted a testing phase with five persons of differing profiles to ensure adequate understanding in French and subsequently presented it to four health professionals to confirm its relevance to the target audience.

#### 2.2.2 The interview guide

The form and substance of the interview schedule were approved by a medical anthropologist with a PhD, specialized in the field of cancer research. To begin with, the guide consisted of the following open prompt: “*Please tell me about the management of addictions in cancer patient care*.*”* The interview was organized to assess how health professionals defined MI and motivational positioning, addictions and their management in the cancer care pathway. Each interview was conducted in a single session, individually, in a quiet office, with the schedule organized to ensure the professional’s full attention.

### 2.3 Data collection phase

Data was collected from October to November 2018. Interviews were conducted by the first author. She is an MSc nurse, a PhD candidate, trained in qualitative research, with no previous association to the caregivers interviewed. The framework in which the collection took place was explained to each of the participants, ensuring their freedom of speech, data protection, anonymization, and a policy of non-reporting to their hierarchy. All interviews took place in a calm office, face-to-face, and were recorded in their entirety on a digital audio recorder. They were then transcribed in verbatim form on a word processing program by a professional secretary. The objective was to collect data until saturation. The average duration of each interview was 22 minutes.

The sample consisted of 15 cancer care providers, mainly oncology nurses. The sample represented a wide variety of ages, seniority and experience. It included nurses working in oncology or hematology in different departments and contexts, ranging from day hospitalization or full conventional hospitalization to interdisciplinary positions (nurse navigators) or the sterile services sector or the hematology transplant department. Although a minority, the sample also included other health professionals such as a tobacco specialist nurse, a social worker and an onco-psychologist, working in both departments (Table 1).

**Table 1:** Participants’ characteristics.

N = 15	N (%)
Profession	
Nurses—Oncology unit	5 (34%)
Nurses—Hematology unit	4 (26%)
Navigator nurses	3 (20%)
Tobacco specialist Nurse & other Health professionals	3 (20%)
Age	
[20–45] years old	12 (80%)
[46–65] years old	3 (20%)
Gender	
Female	13 (87%)
Male	2 (13%)

### 2.4 Qualitative data analysis

These data were analyzed in order to generate themes that could subsequently be associated with the Theoretical Domains Framework of behavior change to investigate implementation problems [14–19].

Specifically, we applied the methodology framework proposed by Braun, V. and Clarke, V. [20]:

1. Familiarization with the data. This phase involved reading and re-reading the data to become immersed in and intimately familiar with its content.
2. Generating initial Codes: this phase involved generating succinct codes that identified important features of the data that might be relevant to answering our research question. It involved coding the entire dataset, and then collating all the codes and all relevant data extracts together for later stages of analysis. Nvivo 11 pro software (QSR International) was used to perform the analyses.
3. Searching for themes: the collection of codes was worked on in pairs with the PhD researcher (VRD) to validate a comprehensive interpretation and a grouping of code elements into themes.
4. The resulting themes were contrasted to the TDF categories independently by the two researchers, and then compared and discussed. Items that could not be included in the TDF framework were thematized separately.
5. Finally, a more refined coding was used for each part of the verbatim used to illustrate the themes.

Three themes and eight sub-themes were generated. Nvivo 11 pro (QSR International) software was used to analyze the collected data, performed by the first author. Once completed, this analysis was discussed in detail with the second author, a medical anthropologist with a PhD, specialized in the cancer research field. The participants did not provide any feedback on the results.

### 2.5 Ethical considerations

The study protocol was approved by the Ethics Committee of the Saint Etienne University Hospital, France (IORG0007394/ N° IRBN712018/CHUSTE). All participants were given written and oral information about the study and gave informed consent to participate. The COREQ checklist guided the preparation of this manuscript [21].

#### 2.5.1 Ethics approval

All procedures performed in studies involving human participants were in accordance with the ethical standards of the institutional and/or the national research committee (Ethics Committee of the Saint Etienne University Hospital, France. NCT03706937) and with the 1964 Helsinki declaration and its later amendments or comparable ethical standards.

#### 2.5.2 Consent to participate

Informed consent was obtained from all individual participants included in the study.

#### 2.5.3 Consent for publication

Informed consent was obtained from all individual participants included in the study.

### 3. Results

The three main themes emerging from the qualitative analysis were (i) representations about addictions-guided care (ii) a difference in practice that depended on the role in care (iii) Motivational Interviewing and motivational positioning driving patient-centered care.

An overview of the different major themes and sub-themes are presented in Table 2.

**Table 2:** Overview of the major themes and sub-themes.

Managing patients treated for cancer and suffering from unresolved issues of addiction			
Theme	Representations about addictions guided care	A difference in practice that depends on the role in care	Motivational Interviewing and motivational positioning driving patient-centered care
Sub-theme	• More complex patients • Cancer seen as a burden • Lack of awareness on smoking cessation	• A taboo of alcohol and toxic addiction • The curative approach—cancer centered • Motivational positioning, at the margin of current practices	• The contributions of motivational positioning to cancer care • A need for training for a patient-centered approach emerging among nurses

### 3.1 Representations about addictions-guided care

#### 3.1.1 More complex patients

For the majority of nurses, patients with alcohol addiction were described as "somewhat uncommon patients,” “Always more complicated than other patients!" or "those kinds of patients.” They were perceived as apart. Most nurses believed these patients required more time and that an effort had to be made to avoid confrontation, language hostilities or upsetting the patient.

*"For us*, *it is complicated to treat patients who have an ear*, *nose or throat cancer and who still have addiction issues; it is difficult for the treatment I give them* (…) *they are somewhat uncommon… they are non-communicative*, *they do not talk to us or they are verbally a bit aggressive* (…) *It is not always easy to give care; they refuse treatment*.*" (Nurse)*

For the onco-psychologist, anchored in a psychoanalytical approach like his or her colleagues, patients with an unresolved addictive disorder were viewed as more vulnerable than patients already weakened by cancer. They described complex care management without behavioral changes in the patients in question.

*"Addiction*, *which is added on top of cancer* (…) (is an) *exacerbating factor or a vulnerability*.*" "They represent two major entities*: *addiction and cancer*. *Together*, *they make for a particularly complex situation*.*” (Onco-Psychologist)*

#### 3.1.2 Cancer seen as a burden

Cancer remained central to concerns. The patient was seen as stupefied by the announcement of a cancer diagnosis. Caregivers perceived it as a unique disease compared to other care pathways.

*"Cancer is a very heavy disease*.*” “Patients suffering from cancer are in a state of stupefaction* (…) *they have just been told that they have cancer*, *which defines the specificity of care*.*" (Nurse)*

Nurses described the diagnosis of cancer as representing a drastic change in the patient's life. They explained that as soon as the disease was announced, patients became psychologically affected, due to the intensity of emotions and upheaval it caused. They also mentioned that the entire daily life of the patient required a reorganization; that these sudden changes were transformations for the body, but also for the mind of the patient.

*"So, in fact, the specificity is that you have to give them time too*! *They have so much information, that their brain cannot do everything at the same time*! *This is the difference, this stupefaction*!" *(Nurse)*

Patients are often seen as very tired, psychologically and physically.

*"There are also patients who are not doing well, they have cardiac issues*, *so this must be mentioned too! There are patients with advanced, already metastatic diseases*, *with a full range of symptoms*.*” (Nurse)*

For patients with an advanced stage of the disease, the concept of project and future projection was modified. Nurses struggled to cope with unsolved addictive disorders associated with cancer, since the cancer was an advanced state and the patient’s life span uncertain. Nurses felt the patient was accumulating burdens.

*"I think it's the cancer, on top of the addiction, that makes the problem harder, it adds an additional burden, anyway*! *And afterwards, the prognosis is often short-term, often*… *for conditions that are a bit particular, anyway!" (Nurse)*

Managing a cancer patient’s addiction presented a challenge for nurses, who felt like they were overloading the patient already dealing with cancer.

*“We are going to ask people to make an extra effort*, *when they are already facing cancer*. *So*, *it’s true that it can actually be complicated*. (…) *Cancer is a whirlwind that turns everything upside down*, *and yet we are asking that of them too*! *It’s the part about adding something more again*!*” (Nurse)*

#### 3.1.3 Lack of awareness of smoking cessation

The tobacco specialist nurse emphasized the overused image that nurses and doctors had of their role in the care. Caregivers appeared to pose a major obstacle to specific support for patients with unresolved addictive disorders.

*“We have several barriers to seeing all the patients*; *the barrier of the caregivers, in any case*!*” (Tobacco specialist nurse)*

For many nurses, managing tobacco addiction was tantamount to total abstinence, which explained why they found it hard to understand the modalities of management based on a reduction in consumption associated with a prescription for nicotine replacement (in this case, patches).

*“It won't change anything if they stop* (…) *Once they have met the tobacco specialist nurse*, *there is not always a change in the patient—some of them will still go outside and smoke their cigarette*, *like they did before meeting her*. *They tell us that they have an agreement with the tobacco specialist nurse and that they can smoke*, *even with the patches*, *despite things like that*, *yes*!*” (Nurse)*

For the other staff members, the opposite was true. They held the conviction that the total abstinence approach of the tobacco specialist nurse caused conflict and hurt the patient, forcing him/her to stop smoking when cancer had just been diagnosed. The tobacco specialist nurse insisted that it was vital to treating physical dependence in the cancer care pathway.

*“Addiction is not only about wearing the patch* (…) *Sometimes*, *we can be sure of temporary abstinence; a patient who has cancer and who is confined to his/her bed—it is not normal that the person suffer from abstinence*.*” (Tobacco specialist nurse)*

The care management strategies of this specialist opposed the representations of his peers, not trained in the specific management of addictions.

*"We have the specificity regarding the staff, where we have quite a few obstacles to overcome, in any case*. *Some experience our intervention as something ‘violent’ regarding the patient who has just been diagnosed with cancer*. *For some, it’s not obvious." (Tobacco specialist nurse)*

### 3.2 A difference in practice depending on the role in care

#### 3.2.1 A taboo on alcohol and drug addiction

For the majority of the sample participants, tobacco was the addiction addressed the most often. Alcohol came in second place but was seldom discussed in a meaningful way. Drug addiction was only very briefly brought up by the three professionals (n = 3: 1 psychologist and 2 caregivers).

*"There are drugs*. *People who take drugs* (…) *I don’t know if it’s not too taboo because we don’t talk about it much*.*” “It can apply to alcohol as well and opiates can be even more taboo; we don’t even talk about it*. *Ultra-taboo*.*" (Onco-psychologist)*

For sterile containment units, addictions seemed inexistant, with the exception of tobacco, which remained questionable for nurses, who lacked a rational explanation.

*"Frankly no*, *it's very strange* (…) *no*, *curiously*, *we don't have people*, *I mean*, *I have come across very few in our department*. *Whereas*, *when you go to other departments*, *you will find some*! *But not in our department*!*" (Nurse)*

Nurses pointed out that the consideration of dependence was directly related to the spontaneous reporting of the patient. For example, in the case of a young opioid-dependent patient (as stated in the medical record), nothing had been implemented, since she had not declared or requested anything.

*“There was nothing specific about opiates*. *There weren't any things implemented at that time*. *She wasn't describing a dependence, but there too, they have to be able to say they're dependent! (Nurse)*

However, the nurse was obliged to collect information from the patient to fill out the medical file. The majority of nurses were uncomfortable with how they approached the addiction subject with the patient. When the nurse broached the topic, it was in a mode that emphasized thoroughness. Otherwise, as seen above, the nurse waited for the patient to declare unresolved issues of addiction on his/her own.

*“Because*, *it’s always taken badly when I ask this question*, *because it means I put the word ‘addiction’ on it* (…) *It has a pejorative side*, *I find* (…) *I don’t know how to ask the question in another way*.*” (Nurse)*

The interview was described as containing leading questions, essentially akin to an interrogation, with the computer as an intermediary, employing closed questions that required binary answers of yes or no. The patient therefore answered these questions of the nurse, without the possibility of speaking about other areas, problems or individual priorities.

*“To know the patient’s priorities, I will ask them the question*. *But it remains a pointed question*; *it is an interrogation.” (Nurse)*

Thus, the directive nursing interview, employing closed questions, like an inventory checklist, seemed to protect the nurse from having to raise questions on sensitive subjects such as the consumption of or addiction to alcohol or other illicit substances.

*"For me*, *it's easier to ask* (…) *like a quote-unquote ‘inventory checklist*,*’ because falling into judging* (them) *I find difficult* (…) *To avoid being judgmental*.*” (Nurse)*

#### 3.2.2 The curative approach: Cancer-centered

For nurses, the fact that the patient continued to consume tobacco while managing cancer did not change the standards of care. The caregiver did not invest heavily in addiction.

*"Afterwards, I'm not going to make any specific adjustment in treatment*, *because there is no real point* (…) *I realize that we haven’t done anything concrete about it." (Nurse)*

However, an exception could be made for the sterile hematology sector. Indeed, nurses explained that these patients in sterile isolation rooms had no choice but to avoid the tobacco or other drugs. Abstinence appeared to be an easy “action,” since the patient could not take any other action, given the hospitalization conditions: complete hospitalization over several weeks without the possibility of leaving the sterile room.

*“Afterwards, I’d say that sterile hospitalization is easy, because, all of a sudden*, *they don’t have a choice*!*” (Nurse)*

According to the nurses, the medical oncologists seemed to focus only on the treatment aspect of cancer. Other ailments, such as pain or fatigue, did not appear to have a place in starting care. It was therefore not uncommon for the patient, experiencing a certain level of pain, not to go to his or her treatment appointment. The medical approach remained pathological-centered and left no room for a holistic approach of the person.

*“For example*, *the doctor*, *what is incredible is that he will only be interested in chemo*! *We have the impression that chemo interests him more than the patient*! *And it’s a bit complicated*! (…) *Later on*, *we sometimes realize there is a problem with pain*, *and that’s why the patient didn’t come; but the problem wasn’t handled on the first day because it wasn’t the question of the day*! *For the doctor*, *the question of the day was to know whether the patient had chemo or not*!*” (Nurse)*

Addictions were added as an additional burden (although they often existed prior to the cancer), with the cancer remaining the central concern, directing management toward a disease-centered approach rather than a patient-centered approach. In most cases, nurses were helpless in the face of cancer-treated patients with unresolved issues of addiction. Cancer care was the priority of the institution and the healthcare teams working there. Therefore, unresolved issues of addiction were not high on the nurses’ list of responsibilities, even if it was sometimes one of the patient's priorities.

*"We are treating a cancerous disease; we do not treat addiction*. *Addiction is not the priority; I’d say that, even for me, it is not my priority*, *whereas sometimes it is the priority for the patient!” (Nurse)*

This is probably the reason why nurses pointed out the absence of smoking prevention in the hospital.

*“It is true that the team doesn’t do prevention* (…) *in terms of the establishment… there is not a whole lot of prevention*, *not in the hallways either*.*” (Nurse)*

#### 3.2.3 Motivational positioning at the margin of current practices

We noted one exception: a nurse with experience in treating alcohol and tobacco dependence stood out from his/her peers. This nurse suffered from the lack of consideration and specific management of addictions by his/her colleagues. This nurse managed to identify and create a rapport with the patient to encourage the latter to talk and built trust with patients during their cancer care. This nurse supported patients in their daily lives, while trying to inspire them to change their behavior. This nurse practiced motivational positioning, but the action remained strictly isolated, with no connection or possibility of sharing with other colleagues.

For the tobacco specialist nurse, who was affiliated with another institution, his/her intervention seemed to fall entirely outside the care pathway, again, focused 100% on cancer. This specialist nurse explained that there was no link to overall care, which, in his/her view, influenced the effectiveness of care.

*“The problem of addictology in the cancer treatment facility—I have the impression that we are a pawn among the rest of it (…) Our care is very*, *very*, *very ‘psy’* (psychological). *And the info that we do not have here*, *it’s kind of a pity*!*” (Tobacco specialist nurse)*

The tobacco specialist nurse practiced MI on a daily basis in care. This specialist nurse, trained in this process, saw the benefits of care in terms of results with the patient. The current nursing practice was based on all the fundamentals of MI, utilized to care for patients undergoing cancer treatment and suffering from unresolved issues of addiction.

This specialist nurse explained that alcohol was often addressed by the tobacco person, because patients suffering from alcohol addiction found it difficult to admit. In this case, the nurse adopted motivational positioning with regard to alcohol and subsequently directed the patient to the addiction specialist team, who intended to follow up.

*"People have a little trouble confessing their alcohol consumption, so through tobacco*, *we're going to bring up the other products that exist." (Tobacco specialist nurse)*

The Social worker informed us of training received in conducting social interviews in the course of his/her studies. Mastering the social interview technique was a requisite for obtaining the degree. The foundations of MI provided the framework that guided the social interview: an interview for and with the patient, to bring out the patient’s motivations for change. The Social worker stressed the need to adopt an empathetic posture, rely on reflective listening and use reformulation and enhancement. Respect for the patient’s rhythm was central to the professional’s approach, not acting in the patient’s place. The formulation of hypotheses and the use of the decision-making balance were an integral part of the practice.

*“It is this relational technique that we are asked to use, which combines empathy*, *a patient-centered approach, valorization, reformulation, active listening*.*” (Social worker)*

The Social worker related how, when there was a medical injunction to arrange directive home care, against the will of the patient, it ended in failure, with the patient doing an about-face upon returning home.

*“The person tells me*, *‘No*! *I don’t want it*!*’ But I still arrange everything urgently*, *because the patient will return home*, *which comes with a medical protocol*, *an obligation*, *and we are responsible for their discharge* (…) *The home aides called and said that he didn’t want it*, *you know*? (…) *When we don’t follow the timing of the patient*, *it does* (results in) *this*!*” (Social worker*)

The Social worker highlighted the difference that this directive stance created with the patient, compared to patient-centered care, where the patient felt considered.

*“The patient feels much more considered in terms of his care*, *that’s it, he feels he is receiving care*! *It makes a difference! I see this difference.” (Social worker)*

Almost a majority of nurses had never heard of MI. A third of the participants of the sample attempted to explain representations of MI but did not corroborate its definition and modalities. They always positioned themselves first, with their filters and their care and management priorities, which was in contrast to the posture taken in motivational positioning. Some claimed it was due to their training and educational experience. They mentioned not knowing how to work differently: the nurse is in a perpetual state of motion with the patient; the nurse must always provide a solution or meet a measurable objective nearly instantaneously.

*“People who listen fully- that is not an objective for me*! *I'm not going to play the pop psychologist!” (Nurse)*

The tobacco specialist nurse tried to show other nurses that listening to the patient is the key to managing unresolved issues of addiction. In motivational positioning, listening is a treatment, it is considered care.

*"But it does not prevent us from fighting against this; talking is a treatment, but it's not the same as administering an injection*. *There are degrees of ‘nobility.’” (Tobacco specialist nurse)*

At times when the nurse wanted to help the patient move towards weaning, the nurse quickly found himself/herself in an impasse. Clearly, the posture adopted by the nurse, combined with the way of connecting with the patient and any addictive problems, was met with a resistance to change in the patient. Furthermore, the discourse increased this resistance to modify his or her habits.

*"After a quarter of an hour*, *quickly*, *we were stuck*! *I thought something was missing and I couldn’t see how to get out of this situation*! *I didn't find an opening or anything*, *I didn’t know what to do*! (…) *It was so painstaking for fifteen minutes*! *There was an opening that told me that he was worried about it*, *but I felt blocked and I didn’t see how*! *Then he asked me if I had a lighter*! *I told him no*, *there’s a limit to everything*! *It was too much for me*!*” (Nurse)*

Clinical care remained under the influence of the nurses’ corrective reflex, referring to the tendency to advise patients about the “right” path to follow for good health. Thus, the nurse advised and warned the patient about the dangers of addiction. The nurse only intended to convince the patient that his/her health would improve, and treatments would prove more efficient after the changes, to provide support. Every fact and gesture of the patient was known to each member of the care team. Caregiver treatment was directive or even injunctive.

*“This is what we are trying to do*, *but it just doesn’t work*.*”* (Make the patient’s own motivation emerge and depart from the common discourse). “*I think we are not very good at highlighting the positive*, *we know how to criticize*. *We have a hard time moving forward at the patient's pace as a caregiver*.*” (Nurse)*

For hospitalized patients, nurses claimed they insisted on education. They reiterated that they were obliged to follow up on what the oncologist did because the latter “made a big deal out of it.” The nurses explained to the patient that smoking increased risks that affected the curability of the cancer. They concluded with the patient, “If there are other addictive elements that come into play, we will not be able to cure them!”

Many nurses disclosed that they used the technique of fear to stimulate the weaning process necessary for the hospital environment (complete hospitalization, sterile post-transplant area).

*"My technique is fear* (…) *It is my way of being in contact with this specific domain*.*" (Nurse)*

### 3.3 Motivational Interviewing and motivational positioning driving patient-centered care

#### 3.3.1 The contributions of motivational positioning to cancer care

Those who practice MI and motivational positioning explained that it was the best way to encourage the patient to change. The decision-making scale involved a technique widely used to enable patients to integrate the notion of management of current addictions while undergoing cancer treatment.

*“We are not necessarily going to talk about completely quitting tobacco because often*, *people are scared*, *thinking we are going to forbid them to smoke* (…) *So we’re going to cite all the pros of quitting smoking and all the cons of cigarettes* (…) *Then we are going to show them a rather empathetic attitude*, *we are not going to impose things on them*, *we are going to gently help them come around to quitting*, *which is different* (…) *We evoke the efforts they have made*, *highlight what they have already achieved*, *and in no case are we going to make them feel guilty*.*" (Tobacco specialist nurse)*

Thanks to this motivational support technique, patients were often satisfied and comforted. Non-directive care, with full listening, without guilt or judgement, reassured patients. They could thus move forward at their own pace and take an active part in changing their behavior and habits, if they so desired. The healthcare professional trained in this approach was there to respect their timing and support them in this process.

*“Actively listening to the patient*, (being) *empathetic (…) many people have an idea of the care regarding tobacco that is completely different; often people do not want to see us (…*.*)* (but) *at the end of the appointment*, *they are happy because*, *and this is the point*, *they did not expect our particular approach*.*” (Tobacco specialist nurse)*

#### 3.3.2 A need for training for a patient-centered approach emerging among nurses

For nurses, a need for training was raised—training that would enable them to relate in a relevant and effective manner to patients undergoing cancer treatment and suffering from an unresolved addictive disorder.

*“For starters*, *I don’t have the skills because I have no specific training*.*” “In my opinion*, *we have to come to a realization* (…) (that) *I don’t have the skills; I don’t have the training*.*” (Nurse)*

Nurses justifiably questioned the mirror game between themselves and the patients. The former seemed to be regularly challenged when facing these patients, amassing counter-attitudes or results against productivity, even though they had fervently invested in care.

*“I think if we had training to approach this type of patient*, *it wouldn’t be so bad*! (…) *I wonder if we are showing them the appropriate attitude*.*” (Nurse)*

As the nurses had previously mentioned, they were the product of a training system with a medico-centered approach. It was the nursing posture anchored in the paternalistic and prescriptive attitude, tinged with the corrective reflex described here.

*“I tell myself that there might be a way to approach it in a different manner*.” *(Nurse)*

## 4 Discussion

### 4.1 Care centered on cancer at the expense of addiction management

Hamant et al. conducted one of the few French studies of dealing with alcohol and tobacco addiction in patients undergoing treatment for ear, nose or throat cancer [22]. Less than 1% of patients treated for cancer reported that they were informed about appointments to help with tobacco dependence or possible support in the management of alcohol consumption. Healthcare professionals involved in the cancer care pathways did not seem invested in the opportunity to support patients with this type of care, complementary to the treatment of their cancer [22]. The professionals interviewed in this qualitative study corroborated these facts. Caregiver management was central to cancer and did not include the possibility that the patient could also be suffering from unresolved addictive disorders. However, the Oncology Nursing Society (ONS) has shown that the management of tobacco addiction has been essential in oncology nursing. It has also established that the role of the nurse in the cancer care pathway is primordial in supporting patients who are experiencing withdrawal or who must wean themselves from their tobacco addiction [23]. However, smoking cessation programs have only recently been integrated as an essential part of cancer management. Active smokers treated for cancer have had poorer outcomes (reduced survival rates and weaker responses to cancer treatments) than those who were able to quit [24]. Results from this ancillary study showed that the oncology nurses often felt helpless to manage and support patients with unresolved addictive disorders. Recent studies have highlighted the need to integrate comprehensive patient care into cancer care pathways. Dauchy and Curé explained that this involves the systematic detection of the patient's vulnerability criteria, to include him/her in a risk group. This would strengthen the care process. One of the criteria was the search for persistent risk behaviors during cancer disease [25]. For this, as evidenced by the findings of this study, a change in the representations of caregivers on the patient treated for a cancer would be necessary. Nurses would also need to change the way they connect with the patient to be able to manage them more holistically, in a patient-centered manner of care, throughout the cancer care pathway.

### 4.2 The need to change practitioners’ attitudes to enable them to provide appropriate care to patients

In France, the paternalistic cultural model of care was the predominant approach until recently. The sensitive relationship often remained characterized by an asymmetrical relationship. This aspect of the nursing position was clearly highlighted in this study. Nurses were often challenged because of their relationship with the patient. This relationship was not one of equality. Conversely, the patient-centered approach aimed to foster patient engagement by placing the caregiver and the patient in a more symmetrical relationship that encouraged the patient to be able to express himself/herself freely, without fear of judgement, on subjects of importance to him/her. Nurses tended to be locked in a pattern of their traditional role and found it decidedly difficult to connect with the patient differently [26]. The results of this research have supported what has been highlighted in the literature. It is well known that the traditional mode of communication focused on a directive approach to delivering information and advice is becoming less effective in maintaining change and significantly changing behavior [5]. Studies have shown that when doctors provided their patients with useful health information, while responding to their emotions, patients experienced a sense of self-control and greater hope. This has had a directly positive impact on their quality of life as well as their survival [27]. The method of entering into a relationship with the patient has therefore proved decisive, with the possibility that the caregiver could let the patient engage and be involved in his/her own care continuum.

Barello et al. explained that nursing was the key to patient engagement. This commitment was associated with patients’ perceptions of the positive attitudes of healthcare professionals on their self-care behaviors. This focused on the essential role of nurses in charge [28]. It also appeared that patients tended to no longer actively be actors in their care when they did not feel they were in a symmetrical relationship with the caregiver, due to a lack of information about their state of health, or as part of a paternalistic attitude from the care system [28]. Our results have demonstrated that nurses who were not trained in motivational positioning struggled to avoid judging patients. They were also expected to obtain concrete results in the management of withdrawal symptoms or to connect with patients affected by addictive behaviors. Some postures adopted by nurses created a "relationship gap" in the caregiver-patient relationship. The majority of nurses interviewed reported that it was difficult to care for patients suffering from addictions vis-à-vis the traditional way nurses treat patients undergoing cancer care. Certain people questioned this issue of communication with patients but were unable to find a response. Michiels related that this relationship gap was the result of attitudes of care such as directional postures, admonitory speech, warnings, reasoned persuasive attitudes, administering advice or even using moralizing words [29]. Situations of a relationship impasse (such as those described in a verbatim where the nurse could not find a suitable angle to help the patient out of ambivalence to discussing smoking) were extremely stressful for the caregivers. They experienced feelings of helplessness and powerlessness in their efforts to pursue care goals. Motivational positioning and its “philosophy” would be a relevant tool to enable nurses to approach the care of patients who suffer from unresolved issues of addiction from another perspective.

### 4.3 The advantages of motivational techniques in the cancer care process for patients suffering from unresolved addiction problems

The motivational techniques employed by two of the health professionals in the sample helped support the patient’s self-esteem (for example, a feeling of self-efficacy) and elicited, among other things, his/her active participation. As Benarous et al. pointed out, a professional caregiver trained in MI did not experience an increase in his/her appointment time or frequency [5].

However, this approach is still not well known in France outside the sphere of addictology, which corresponds to the results of this research. According to Ong et al, MI has a prime role to play in these intervention programs in order to enable patients to solve their issues of ambivalence and identify barriers to smoking cessation, among others. It is recommended that the first interview be held as close as possible to the announcement of the cancer diagnosis; the moment of diagnosis is favorable to the promotion of smoking cessation. The second most suitable time is during the patient's hospitalization. In both periods, patients have increased interaction with healthcare professionals. Nurses can support them in the quest to quit smoking [24].

Furthermore, as our results have illustrated, most of the nurses we met believed that the patient needed to be protected during this period. They held such a belief almost certainly because of their representations on the effects of the cancer announcement on the psychological sphere of the patient. They described a patient who is stupefied and profoundly stricken emotionally. This perception did not engage them in a patient-support approach to addressing any addictive disorders in progress. They felt they were accompanying the patient, which put them at risk in their own nursing role. On the other hand, for professionals trained in motivational positioning, the approach greatly differed. Their care was adapted to the fragility generated by the announcement of the cancer diagnosis. Still, according to Ong et al., the majority of patients undergoing cancer treatment who are still active smokers generally fail to treat it themselves. Many would like to quit, but the majority still smoke. A combination of MI and substitution treatments, initiated as close as possible to diagnosis, is more likely to make withdrawal permanent [24]. This demonstrates the amount of work that remains to be done with medical and nursing teams managing patients treated for cancer and suffering from addictive disorders, in order to change their representations in terms of times conducive to intervention (of smoking in particular). With regard to tobacco, overall care must be organized at all levels of the care pathway, including actions to prevent and manage smoking in cancer care facilities [25]. These recommendations should be incorporated into good cancer practice, in accordance with the latest guidance from several organizations such as the American Society of Clinical Oncology (ASCO) and the Association for Cancer Research (AACR) [30].

Developing patient-centered care on the care pathway in oncology is necessary to be able to take account of unresolved addictive disorders, where they exist. A paradigm shift is needed to improve communication between healthcare professionals and patients. Motivational positioning is a posture of care that promotes this communication, in addition to supporting his/her empowerment. This research suggests the need to adapt the cancer care pathway in order to integrate the necessary care for patients who concomitantly suffer from unresolved addictive disorders.

#### 4.3.1 Limitations of the study

The results of our research should be compared with similar studies at other cancer treatment facilities. The sample did not include ‘advanced practice nurses’ because, as a new profession, French establishments generally do not employ them. We suggest that research should be pursued on a wider scale, in several comprehensive cancer centers, when ‘advanced practice nursing’ becomes more prevalent in cancer treatment facilities to collect information for further findings.

The small size of the sample cannot be used to extract representative quantitative statistical data due to lack of power. However, the sample size is compatible with reliable qualitative analysis [31].

## 5 Conclusions

It is becoming essential in France that nurses change their practices to support patient engagement in the management of unresolved addictive disorders along the oncology care pathway. This must involve strengthening their training in this domain, specifically the dissemination of MI techniques to support patient motivation, precisely considering the traditional positioning of nurses who have acquired counterproductive effects. The results of this ancillary study illustrate that such improvements are not yet integrated into French standard practices in oncology. The generalization of the MI and its tools, in current oncology practice, both in terms of physicians and nursing teams, would make it possible to improve the management of addictions in a patient-centered approach, support the empowerment of patients in the cancer care pathway and promote positive communication between caregivers and patients.

## Supporting information

S1 Checklist COREQ (COnsolidated criteria for Reporting Qualitative research) checklist. (PDF)

## Abbreviations

MI: Motivational Interviewing
TDF: Theoretical Domains Framework
ONS: Oncology Nursing Society
ASCO: American Society of Clinical Oncology
AACR: Association for Cancer Research
